# Amelioration of lithiatic injury to renal tissue by candesartan and sodium thiosulfate in a rat model of nephrolithiasis

**DOI:** 10.1371/journal.pone.0251408

**Published:** 2021-05-13

**Authors:** Nahla E. El-Ashmawy, Hoda A. El-Bahrawy, Heba H. Ashmawy, Eman G. Khedr

**Affiliations:** Department of Biochemistry, Faculty of Pharmacy, Tanta University, Tanta, Egypt; University of Messina, ITALY

## Abstract

**Aim:**

Nephrolithiasis is a chronic metabolic condition affecting 10% of population worldwide. The present study aimed to investigate the possible protective role of candesartan (CAND) and sodium thiosulfate (STS) in ameliorating ethylene glycol (EG) induced nephrolithiasis.

**Methods:**

One hundred male Wistar rats were divided into five groups: Normal control group, nephrolithiasis (EG) group (1% EG in drinking water), Cystone (CYS) group (EG + 750 mg/kg CYS, orally, once daily), STS group (EG + 0.4 gm/kg STS, intraperitoneally, 3 times/week) and CAND group (EG + 70 μg/mL CAND in drinking water). Treatments and EG administration commenced on the same day and continued for 28 days. CYS was used as reference drug. Urine, blood, and renal tissues were collected at the end of the experiment for assessment of kidney function tests (serum creatinine and urea), urinary (8-hydroxydeoxyguanosine (8-OHdG), calcium and oxalate), inflammatory and oxdative stress biomarkers (transforming growth factor beta (TGF-β), osteopontin (OPN) and ratio of reduced glutathione to oxidized glutathione (GSH/GSSG)) in renal tissue.

**Results:**

Serum (creatinine and urea), urinary (8-OHdG and oxalate) and renal (OPN and TGF-β) were significantly reduced in CAND and STS groups compared to EG group. Furthermore, renal GSH/GSSG and urinary calcium were significantly increased in CAND and STS groups compared to EG group. Histopathological results support the biochemical findings; CAND and STS groups showed less retention of crystals and necrotic damage in kidney. Also, microscopic examination of urine revealed less crystal for CAND and STS groups.

**Conclusion:**

Candesartan and sodium thiosulfate exhibited protective effect against nephrolithiasis.

## 1. Introduction

Nephrolithiasis is a public disease affecting about 10% of the world population and is considered one of the most painful disease. The prevalence has elevated over the preceding several years due to altering dietary habits, increasing obesity, and global warming [1]. The recurrence rates of nephrolithiasis rise to 50% of stone patients in epidemiological studies [2, 3].

Nephrolithiasis is accompanied with declined kidney function and with chronic kidney disease [4]. This disease may cause severe medical consequences such as obstruction, hydronephrosis, infection and hemorrhage in the urinary system if left untreated. Stones are mostly calcium comprising stones, specifically calcium oxalate (CaOX) (80%) and other types are 20% [5]. Surgical operation, lithotripsy and local calculus disruption using high-power laser are popular treatment options to eliminate the calculi. However, surgical procedures are related with severe complications such as hemorrhage, infection, acute renal injury, tubular necrosis, and afterwards kidney fibrosis, declined renal function, ureteral perforation [6]. Fragmentation procedure (lithotripsy), despite of being minimally invasive, is associated with amplified stone recurrence rate and high cost [7, 8]. Hence, it is urgent to search for new strategies for treatment and prevention of recurrent kidney stone disease.

Although the definite mechanism of stone development is not entirely understood, oxidative stress is confirmed to have an obvious role in the development of nephrolithiasis [9, 10]. Reactive oxygen species (ROS) are generated through the interaction between oxalate/calcium oxalate monohydrate (COM) crystals and renal cells and are blamed for the several cellular responses by stimulating nicotinamide adenine dinucleotide phosphate (NADPH) oxidase which is a significant source of ROS in kidney cells during stone development. Consequently, drugs that reduce oxidative stress are good candidates to prevent nephrolithiasis. In particular, sodium thiosulfate (STS), an FDA-approved drug used to reduce adverse reactions to cisplatin and in the emergency treatment of cyanide poisoning, has been proposed as a potential antiurolithiatic agent due to its electron donation and hunting potential for free radicals [11].

In addition, oxalate and COM trigger the renin–angiotensin system (RAS) [12]. Inflammatory response and oxidative stress are augmented by local angiotensin production. Renal cell injury and ROS stimulate transforming growth factor beta (TGF-β) secretion [13]. TGF-β, profibrotic cytokine, is up-regulated in chronic kidney diseases leading to glomerulosclerosis and tubulointerstitial fibrosis. Different types of renal cells go through diverse pathophysiological changes induced by TGF-β, leading to apoptosis and hypertrophy, which eventually result in renal dysfunction [14].

Osteopontin (OPN) is a chemoattractant which prevents mineralization by drawing inflammatory cells to location to eradicate crystal formation. Thus, it results in renal damage by phagocytic NADPH oxidase stimulation and free radical development, and hence OPN is related to progressive kidney injury [15].

Compromised kidney function is more predominant in patients with urolithiasis than those without stone development. The pathophysiology of hypertension and compromised renal function are closely related. Patients with kidney impairment have shown an acid–base homeostasis disruption, salt and water handling disturbance, which in turn drives hypertension [16]. Likewise, effects of elevated arterial blood pressure can directly hurt renal parenchyma and vasculature, thus resulting in kidney damage. It is also broadly established that hypertension is more prevalent in patients with calcium urolithiasis than the normal people [17].

The angiotensin receptor blocker used as blood pressure-lowering agent candesartan (CAND), has also been proposed as an antiurolithiatic agent. It was reported that the kidneys of hyperoxaluric rats treated with candesartan, showed less calcium oxalate stones, and reduced oxidative stress biomarkers [18]. Indeed, CAND has been shown to protect the kidney from oxidative stress injury by inhibiting reactive ROS generation and tubulointerstitial inflammation. Furthermore, it has shown significant role on stone development in the kidney by lowering NADPH oxidase and TGF-β levels [19].

The aim of the current study was to investigate the possible protective effects of candesartan and thiosulfate against nephrolithiasis and to elucidate the underlying biochemical mechanisms. Nephrolithiasis was induced by ethylene glycol (EG). In this model, EG is metabolized to glycolate, glyoxylate and oxalate, which leads to calcium oxalate crystals (monohydrate (COM) and dihydrate (COD)) development in both urine and the kidneys along with tubular damage, dilatation and interstitial inflammation [20]. As a positive control [21], we used the antiurolithiatic drug Cystone which is herbal formulation used to treat and preclude urinary tract infection and kidney stone formation [22, 23].

## 2. Materials and methods

### 2.1 Animals

The study was conducted in accordance with guidelines for care and use of laboratory animals, approved by Research Ethics Committee of Faculty of Pharmacy, Tanta University, Egypt (FPTU-REC,141\2013\970). One hundred Wistar male albino rats (190–230 g) were housed in wire cages under controlled environmental conditions and allowed standard rodent chow *ad libitum* and free access to tap water for 2 weeks for acclimatization before the experiment.

### 2.2 Experimental design

After 14 days of acclimatization rats were randomly divided into five groups (number of animals (n) = 20/group) according to the treatments as follows: Normal control (NC) group: Rat was maintained on water *ad libitum*. Those in the other four groups were maintained on 1% EG in drinking water, as described by [24]. One of the four groups was used as the nephrolithiasis control (EG) group. Rats in the Cystone (CYS) group received 750 mg/kg CYS once daily by oral gavage [25], rats in the sodium thiosulfate (STS) group received 400 mg/kg STS 3 times/week intraperitoneally (i.p.) [26], and rats in the candesartan (CAND) group received 70 μg/mL CAND in drinking water [19], in parallel with EG. Treatment in the CYS, CAND, and STS groups, and EG administration in the nephrolithiasis group were started on the same day and continued for 28 days.

### 2.3 Sample collection

At the end of the experiment, urine samples were collected using metabolic cages, then centrifuged to remove any debris and kept at -20°C. Afterwards, blood was withdrawn *via* cardiac puncture and serum was collected and kept at -20°C till biochemical measurements. Rats were sacrificed by cervical dislocation and kidneys were detached instantly. Right kidneys were preserved in 10% buffered formalin solution at room temperature for histopathological studies. Kidney homogenate samples were prepared from left kidneys and stored at -80°C for biochemical analysis.

### 2.4 Analysis of urine samples for crystalluria

For microscopic analysis, one drop urine sample was spread on glass slide and blindly examined under light microscope. Number of oxalate crystals was counted in multiple samples from each group and the mean crystal number for each group was calculated [3].

### 2.5 Determination of kidney function biomarkers

Serum urea and creatinine were assessed colorimetrically using commercial kits obtained from (Biodiagnostic^®^, Egypt). Serum urea and creatinine were expressed in mg/dL. The serum urea level was measured colorimetrically according to the urease-Berthelot method [27] and the serum creatinine level according to the method described by Slot [28].

### 2.6 Determination of urinary 8-hydroxydeoxyguanosine, oxalate and calcium

Urinary 8-hydroxydeoxyguanosine (8-OHdG) concentration was measured using rat (8-OHdG) enzyme linked immunosorbent assay (ELISA) kit (Sunred Biological Technology^®^, Shanghai, China) according to the manufacturer protocol. Urinary 8-OHdG concentration was expressed as ng/mL urine. Urinary oxalate concentration in μM was estimated according to the colorimetric method described by Zuo *et al*. [29] using an oxalate colorimetric assay kit (BioAssay systems^®^, USA). Urinary calcium concentration in mg/dL was estimated according to the colorimetric method described by Kessler & Wolfman [30] using a colorimetric kit (Spectrum Diagnostics®, Egypt).

### 2.7 Determination of transforming growth factor-beta (TGF-β) and osteopontin (OPN) in kidney tissues

Tissue samples were cut, weighed and homogenized after adding phosphate buffered saline (pH 7.4) by tissue homogenizer. Supernatant was collected after centrifuging for 20 minutes at 3000 rpm for biochemical measurements. Renal TGF-β and OPN concentrations were measured using rat TGF-β and OPN ELISA kits (Sunred Biological Technology^®^, China) according to the manufacturer protocol. Renal TGF-β concentration was expressed as pg/g tissue. Renal OPN concentration was expressed as ng/g tissue.

### 2.8 Determination of ratio of reduced glutathione (GSH) to oxidized glutathione (GSSG) in kidney tissues

Renal GSH and GSSG concentration was measured using rat GSH and GSSG ELISA kits (NOVA^®^, Beijing, China) according to the manufacturer protocol. Renal GSH and GSSG were expressed as pg/g tissue then GSH/GSSG ratio was calculated.

### 2.9 Histopathological examination of kidney tissue

Kidney tissues were cut into 3-μm thick sections using microtome (Leica RM 2135, Germany), deparaffinized and hydrated in descending series of ethyl alcohol. Sections were stained with hematoxylin (H) and eosin (E) stains, dehydrated and cleared in xylene [31]. Then, an expert histopathologist blindly examined the slides using a light microscope (Leica DM 500, Switzerland) and photographed. Six fields for each kidney section were examined and assigned for severity of changes (Renal necrosis, tubular degeneration, inflammation, urolithiasis and fibrosis) using scores of (-: Not detected), (+: Mild lesions), (++: Moderate lesions), (+++: Severe focal lesions) and (++++: Severe diffuse lesions).

### 2.10 Statistical analysis

Data was analyzed with statistical package for social science (SPSS) software. Results are presented as mean ± standard deviation (SD) and as a percent of change. One-way analysis of variance (ANOVA) test was used for statistical judgement among groups using Fisher’s least significant differences (LSD) method for comparison between two groups. Experimental results were analyzed at significance level p < 0.05.

## 3. Results

### 3.1 Effect of different treatments on urine crystalluria revealed by microscopic examination

**Fig 1:**
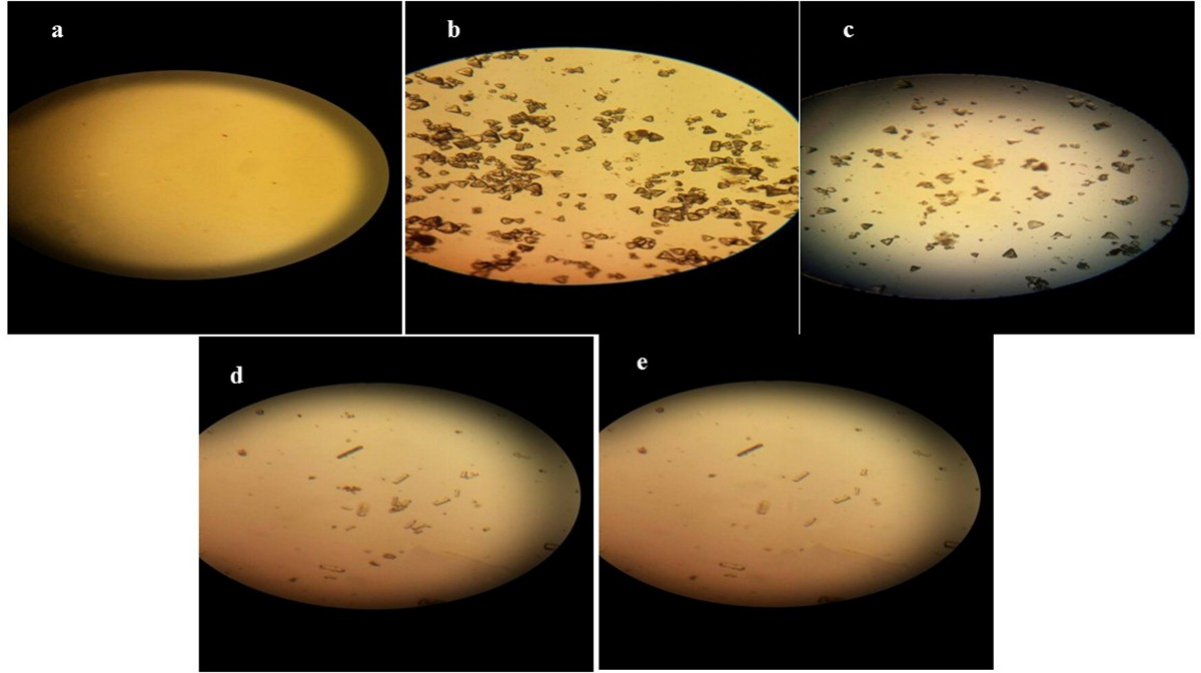
Microscopic examination of urine samples (X200). **(A)** NC group **(B)** EG group **(C)** CYS group **(D)** STS group **(E)** CAND group. NC group: Rats received water; EG group: (1% ethylene glycol in drinking water for 28 days); CYS group: (EG + 750 mg/kg CYS, orally, once daily); STS group: (EG + 0.4 g/kg STS, i.p., 3 times/week); CAND group: (EG + 70 μg/ml CAND in drinking water). Treatments were administered simultaneously with EG drinking water for 28 days.

To study whether CAND and STS prevent nephrolithiasis, we treated with CAND or STS rats receiving 1% EG-supplemented drinking water to induce nephrolithiasis and we evaluated crystal formation in urine. Microscopic examination of urine samples revealed that no CaOX crystals were detected in NC group. However, EG group showed abundant (>100) CaOX crystals of both subtypes; monohydrate (COM) and dihydrate (COD). CYS, STS and CAND treatments significantly reduced the number of CaOX crystals as follows: CYS group showed 85±15 crystals of both COM and COD. STS group showed 76±15 crystals of only COM. CAND group showed 57±23 crystals of COM **(Fig 1)**. These results suggest that STS and CAND treatments result in a reduction in the number of CaOX crystals more than CYS treatment.

### 3.2 Effect of different treatments on kidney function biomarkers

**Fig 2:**
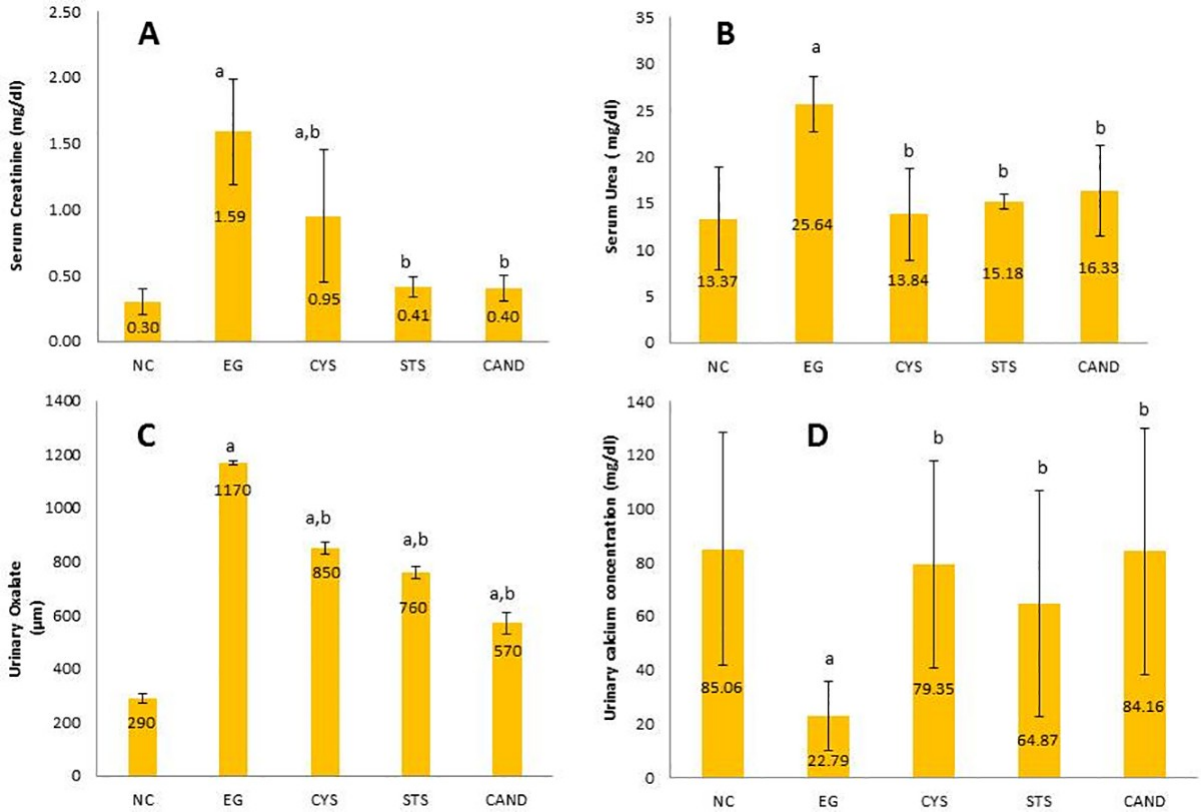
Effect of CYS, STS, CAND treatments on: (A) Serum creatinine (B) Serum urea (C) Urinary Oxalate (D) Urinary calcium concentration. Data are represented as a mean ± SD (n = 20/group), significance was set at *p* <0.05; a: Significant *vs* NC group, b: Significant *vs* EG control group. NC group: Rats received water; EG group: (1% ethylene glycol in drinking water for 28 days); CYS group: (EG + 750 mg/kg CYS, orally, once daily); STS group: (EG + 0.4 g/kg STS, i.p., 3 times/week); CAND group: (EG + 70 μg/mL CAND in drinking water). Treatments were administered simultaneously with EG drinking water for 28 days.

To evaluate the effect of CAND and STS treatments on lithiatic injury we assessed kidney function by measuring serum creatinine and urea. Nephrolithiasis control group exhibited significantly higher serum creatinine and serum urea (430% and 91%, p<0.05), respectively than the NC group. On the other hand, treatment with CAND or STS significantly decreased serum creatinine by 74% (p<0.05) and serum urea by (35% and 30%, p<0.05), respectively compared to EG group to a level that was not significantly different from NC group. Similarly, the CYS group showed significantly lower serum urea than the EG group (50%, p<0.05); however, although the serum creatinine level was significantly lower than that in the EG group (40%, p<0.05), it was actually significantly higher than in the NC group (**Fig 2A and 2B**). These results show that CAND and STS treatments could preserve kidney function better than CYS.

### 3.3 Effect of different treatments on urinary oxalate and calcium concentrations

Rats of EG group showed significant increase in urinary oxalate by 300% (p<0.05) compared to NC group. However, urinary calcium level showed a significant decrease in EG group by 70% when compared to NC group (p<0.05). On the other hand, treatment with CYS, STS and CAND resulted in significantly lower urinary oxalate (27%, 35%, and 51%, p<0.05) respectively compared to EG group. On the other hand, urinary calcium was significantly increased in CYS, STS and CAND groups by 248%, 180%, and 270% (p<0.05) respectively compared to EG group (**Fig 2C and 2D**). These results show that CAND and STS treatments could decrease urinary oxalate and preserve normal urinary calcium level.

### 3.4 Effect of different treatments on renal transforming growth factor-beta (TGF- β) and renal osteopontin (OPN)

**Fig 3:**
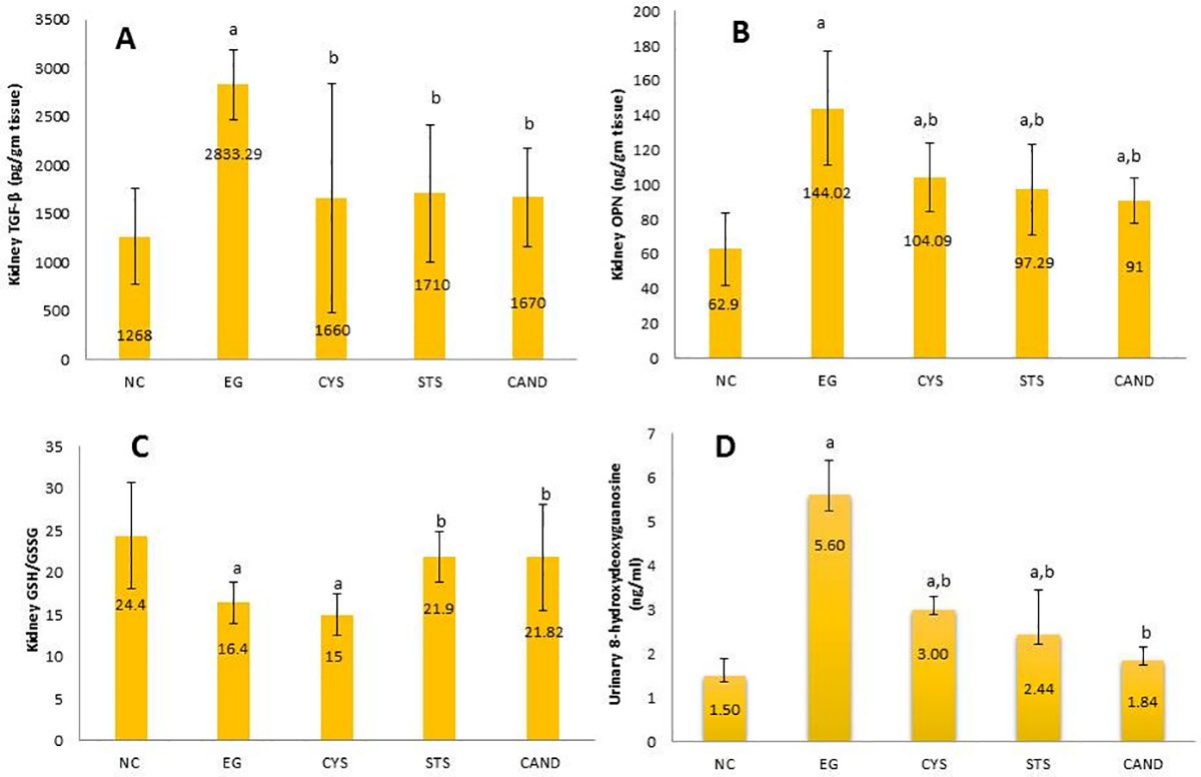
Effect of CYS, STS, CAND treatments on: (A) Renal transforming growth factor-beta (TGF- β) (B) Renal osteopontin (OPN) (C) renal oxidative stress parameter GSH/GSSG (D) Urinary oxidative stress biomarker (8-OHdG). Data are represented as a mean ± SD (n = 20/group), significance was set at *p* <0.05; a: Significant *vs* NC group, b: Significant *vs* EG control group. NC group: Rats received water; EG group: (1% ethylene glycol in drinking water for 28 days); CYS group: (EG + 750 mg/kg CYS, orally, once daily); STS group: (EG + 0.4 g/kg STS, i.p., 3 times/week); CAND group: (EG + 70 μg/mL CAND in drinking water). Treatments were administered simultaneously with EG drinking water for 28 days.

EG group exhibited significantly higher renal TFG-β and renal OPN (120% and 125%, (p<0.05), respectively) than NC group. On the contrary, CYS, STS and CAND groups showed a significant reduction in renal TFG-β by 41%, 40%, and 41%, (p<0.05), respectively compared to EG group to near normal control level. On the other side, renal OPN level was significantly reduced in CYS, STS and CAND groups by 28%, 32%, and 37%, (p<0.05) respectively compared to EG group but still higher than normal control level (**Fig 3A and 3B**). These results show that both CAND and STS have anti-fibrotic and anti-inflammatory effects comparable with CYS treatment.

### 3.5 Effect of different treatments on oxidative stress biomarkers

To evaluate the protective effect of CAND and STS treatments on oxidative stress in nephrolithiasis we measured oxidative stress biomarkers in renal tissue and urine (renal GSH to GSSG ratio and urinary 8-OHdG). The renal GSH to GSSG ratio was significantly lower in the EG group (33%, p<0.05) than in the normal control group. However, the CAND and STS groups showed significantly higher GSH to GSSG ratios by (45%, p<0.05) than EG group. No change observed in GSH to GSSG ratio in CYS group compared to EG group. On the other hand, urinary 8-OHdG in the EG group was significantly higher by (250%, p<0.05) than NC group. Conversely, CYS, STS and CAND groups showed significantly lower urinary 8-OHdG by (46%, 50%, and 70%, p<0.05) respectively than the EG group (**Fig 3C and 3D**). These results show that both CAND and STS have antioxidant efficacy more powerful than CYS that could protect animals from oxidative stress.

### 3.6 Effect of different treatments on renal tissue histology

**Fig 4:**
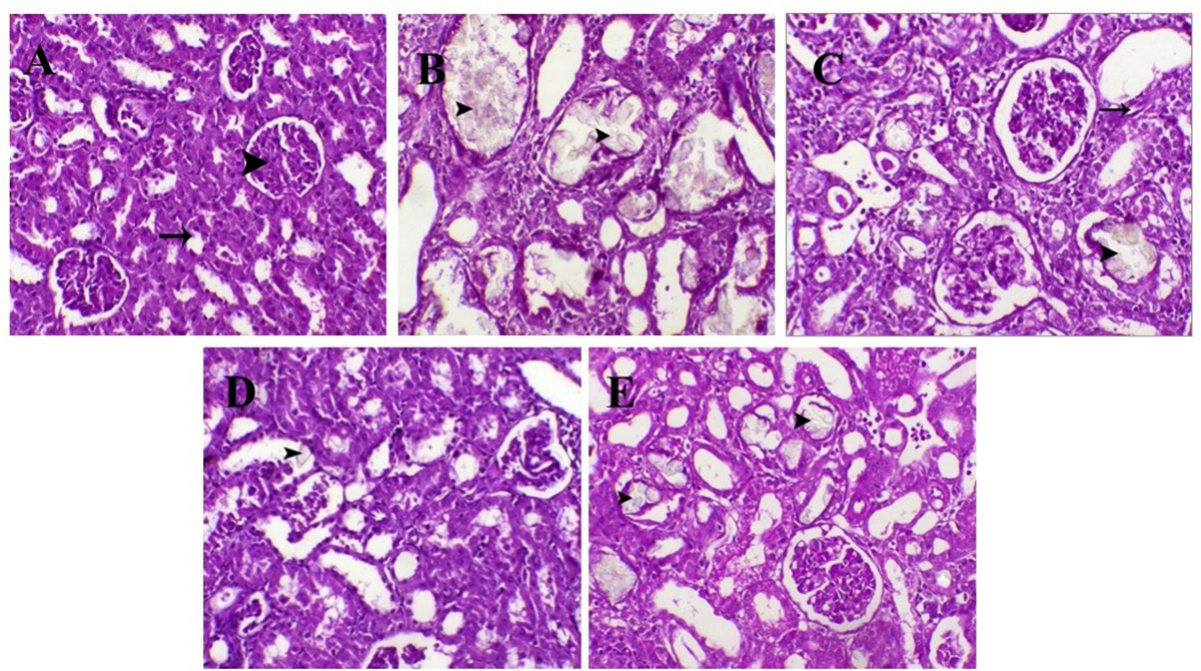
Photomicrograph of kidney tissue sections stained with H&E (X200): **(A) NC group** normal renal glomeruli (arrow head) and tubules (arrow); **(B) EG group** severe tubular degeneration with massive tubular urolithiasis (arrowheads); **(C) CYS group** moderate degeneration (arrow) and tubular urolithiasis (arrowhead); **(D) STS group** mild degeneration and tubular urolithiasis (arrowhead); **(e) CAND group** moderate degeneration and tubular urolithiasis (arrowheads). NC: Normal control; EG: Ethylene glycol; CYS: Cystone; STS: Sodium thiosulfate; CAND: Candesartan.

Kidney sections of control group showed normal histological structure of renal glomeruli and tubules. However, kidney sections of EG group showed severe tubular cell degeneration, necrosis and fibrosis. They also showed moderate inflammation and massive intratubular deposition of crystals. Kidney sections of rats treated with CYS showed moderate tubular urolithiasis with moderate degree of nephritis and fibrosis. They also showed moderate tubular regeneration. On the other hand, kidney sections of STS treated rats showed the lowest level of renal degeneration, necrosis and fibrosis. They also showed moderate tubular regeneration, mild intratubular urolithiasis and nephritis. Kidney sections of CAND treated group showed moderate tubular degeneration, necrosis, fibrosis and intratubular urolithiasis. They also showed moderate tubular regeneration and mild nephritis (**Fig 4)**. Semi quantitative assessment of the histopathological findings within the treated groups is demonstrated in **Table 1**.

**Table 1:** Semi quantitative scoring of kidney lesions associated with urolithiasis in different treated groups.

Groups	Renal degeneration and necrosis	Tubular regeneration	Inflammation	Urolithiasis	Fibrosis
**NC group**	**-**	**-**	**-**	**-**	**-**
**EG group**	+++	+	++	++++	+++
**CYS group**	++	++	++	++	++
**STS group**	+	++	+	+	+
**CAND group**	++	++	+	++	++

NC group: Rats received water; EG group: (1% ethylene glycol in drinking water for 28 days); CYS group: (EG + 750 mg/kg CYS, orally, once daily); STS group: (EG + 0.4 g/kg STS, i.p., 3 times/week); CAND group: (EG + 70 μg/mL CAND in drinking water). Treatments were administered simultaneously with EG drinking water for 28 days. NC: Normal control; EG: Ethylene glycol; CYS: Cystone; STS: Sodium thiosulfate; CAND: Candesartan. (-) no detectable lesions; (+) mild lesions; (++) moderate lesions; (+++) severe focal lesions; (++++) severe diffuse lesions.

## 4. Discussion

Due to the high recurrence rate of kidney stones, the potential use of drugs such as candesartan (CAND) and sodium thiosulfate (STS) to ameliorate nephrolithiasis, and the molecular mechanisms of their actions, are of interest.

Ethylene glycol is commonly used to form nephrolithiasis model in rats to simulate the formation of kidney stones in humans [32]. Nephrolithiasis was evidenced by both microscopic urine analysis and kidney histopathological examination. Deterioration of kidney function was observed in EG group due to accumulation of calcium oxalate crystals in the kidney that causes injury leading to decreased kidney function [33]. Our observations were in agreement with Alenzi *et al*. [34]. However, CAND and STS in prophylactic regimens have protected the kidney from this injury. This was evidenced by serum urea and serum creatinine that were decreased to normal levels. Our results were in accordance with previous studies [11, 35]. Normal serum urea with high serum creatinine was observed in CYS group. According to these results, CAND and STS show better kidney protection than CYS.

Ethylene glycol induction increases serum and urinary oxalate (as oxalate is one of its metabolites) and decreases serum and urinary calcium as calcium is consumed in complexing with oxalate forming calcium oxalate stones [3, 36]. Drugs used in this study corrected the hypocalcemia related to EG induction and reversed low urinary calcium into normal urinary calcium level. As the investigated drugs decease the deposition of calcium oxalate crystals in kidney so, they decrease consuming calcium resulting in higher urinary calcium level [3]. CAND and STS treatments resulted in significantly lower urinary oxalate level compared to EG group. Urine oxalate excretion is controlled by the interaction between oxalate production, intestinal oxalate absorption from foods, and secretion from blood back into the gut lumen. Oxalate production in our study comes from hepatic metabolism of EG and this is not affected by drugs used in this study while intestinal absorption is decreased and secretion from blood back into gut lumen is increased due to the availability of calcium for binding with oxalate in the intestine thus, decreasing oxalate intestinal absorption [37]. Consequently, oxalate level in blood and urine will decrease thus reducing the probability of stone formation.

Impeding inflammation and oxidative stress in the kidneys has been proposed as a prospective approach for the amelioration of kidney stones [38]. In our study, renal GSH/GSSG ratio, oxidative stress biomarker, decreased in EG treated rats due to enormous reactive oxygen species production as a result of hyperoxaluria. These observations were in agreement with Sharma & Naura [39]. In CAND group this ratio was elevated back to the control level. In STS group this ratio was elevated back to near control level and this result was in accordance with previous studies [26, 40].

8-hydroxydeoxyguanosine, oxidatively modified guanosine, can be considered another oxidative stress biomarker. In our study, urinary 8-OHdG in the EG group was significantly higher than in the normal control group, and this result was in accordance with previous studies [3]. In CAND group 8-OHdG was near to its level in normal control showing that CAND has protected renal tubular cells from reactive oxygen species. In STS group 8-OHdG was significantly lower than its level in EG group but still higher than that of normal control.

In the CAND group, the levels of oxidative stress biomarkers were significantly lower due to its role as angiotensin receptor blocker. Angiotensin II (Ang II) increases the production of inflammatory mediators and reactive oxygen species so decreasing its binding to its receptor that decreases its activity might preserve glutathione level and this is was supported by previous studies [41]. In addition, decreasing the actions of Ang II protects DNA from oxidative damage, resulting in a lower level of 8-OHdG.

For STS group oxidative stress biomarkers were diminished due to the antioxidant and chelation power of sodium thiosulfate. STS free radical scavenging power is due to being rich in sulfur thus providing reducing environment that confers renal protection and this result was in accordance with previous studies [11, 26, 40]. Contrary, no significant effect on GSH/GSSG ratio was observed in CYS group but it decreased 8-OHdG significantly to level lower than its level in EG group but still higher than that of normal control. Consequently, CAND and STS treatments show antioxidant efficacy more powerful than CYS treatment suggesting better protective activity against nephrolithiasis.

OPN has several binding sites as integrin sites and calcium ion binding sites, and cells can attach to OPN integrin sites by coupling with multiple integrin receptors on the cell surface. OPN calcium binding sites are inclined to bind to calcium ions in calcium oxalate, and thus calcium oxalate crystal growth and aggregation can be inhibited by free OPN. However, epithelial cells of kidney that can’t be attached with crystals of CaOX can be transformed to renal epithelial cells to stimulate the formation of calcium oxalate stones after being attached to OPN. Therefore, OPN can encourage the development of calcium oxalate nephrolithiasis rather than preventing it [42].

In our study, OPN secretion was significantly higher in the EG group than in the normal control group. The OPN level in the CAND and STS groups was significantly lower than EG group illustrating the potential beneficial effects of using candesartan as a prophylactic agent in nephrolithiasis. Our results are consistent with results from previous studies [42]. OPN synthesis and production was down regulated on AT1 receptor blockage suggesting an involvement of the RAS in hyperoxaluria-induced OPN up regulation. The involvement of Ang II in renal OPN expression has been suggested by previous studies evidenced by extreme proliferation in OPN protein and mRNA levels was identified in renal epithelial cells upon Ang II infusion [43], and Ang II was proposed to stimulate OPN expression in renal cells directly or through the promotion of TGF-β [44]. Blockage of angiotensin receptor by candesartan down regulated OPN synthesis and production. For STS group, its lowering effect on OPN is due to anti-inflammatory activity mediated by lowering TGF-β level [45].

Transforming growth factor beta (TGF-β) is strongly correlated with renal cell injury that occurs during stone formation. In our study, TGF-β level was significantly higher in the EG group than in the normal control group. TGF-β level in CAND group was similar to its level in normal control group. So, candesartan has protected renal cells from deleterious effects of hyperoxaluria. Our results was supported by previous studies [18, 19]. Also, TGF-β level in STS group was near to its level in normal control group showing that STS has protected kidney from injury by CaOX crystals and this was in accordance with previous studies [45].

Hyperoxaluria augments ROS production and accelerates oxidative stress thus triggering TGF-β production and activation and leading to high level of TGF-β in EG group. Angiotensin receptor blockers inhibit TGF-β directly by blocking Ang II which promotes TGF-β expression and activation which may explain the normal level of TGF-β in CAND group [19]. For STS group, decreasing the level of TGF-β is due to the free radical scavenging power of STS, which decreases oxidative stress [40].

The biochemical findings for both CAND and STS groups are supported by the histopathological results, which showed less retention of crystals in the renal tubules and less necrotic damage in renal tissues from both groups. Furthermore, microscopic examination revealed fewer crystals in the urine samples from both CAND and STS groups than the EG group.

**Fig 5:**
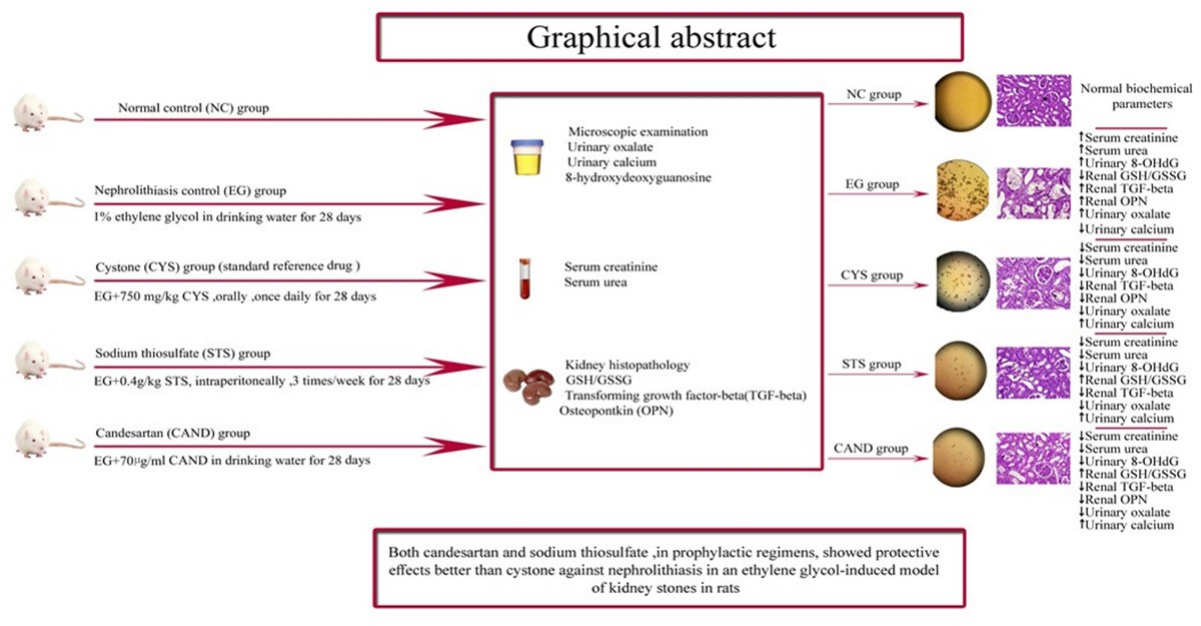
Graphical abstract. NC: Normal control; EG: Ethylene glycol; CYS: Cystone; STS: Sodium thiosulfate; CAND: Candesartan.

Summary of the study design and results is shown in (**Fig 5**).

## 5. Conclusion

In the present study, candesartan and sodium thiosulfate exerted anti-inflammatory, antioxidant effects and protected against calcium oxalate renal calculi formation in EG nephrolithiasis rat model. On the molecular level, both drugs decreased the level of the fibrotic mediator TGF-β, the inflammatory mediator OPN and oxidative stress (clearly observed by high GSH/GSSG and low 8-OHdG). The treatment of either candesartan or sodium thiosulfate in prophylactic regimen could correct the deterioration of renal tissue histopathology and function. Therefore, they could be considered a promising strategy in preventing recurrent kidney stones and nephrolithiasis associated complications. Further prospective studies are needed to investigate whether the observed effects of candesartan or sodium thiosulfate are likely to be achieved in therapeutic regimens. Further studies are also warranted to examine the efficacy of candesartan or sodium thiosulfate in inhibiting the formation of renal calculi in clinical settings.

## List of abbreviations

CAND: Candesartan
STS: Sodium thiosulfate
EG: Ethylene glycol
CYS: Cystone
8-OHdG: 8-Hydroxydeoxyguanosine
TGF-β: Transforming growth factor beta
OPN: Osteopontin
GSH/GSSG: Ratio of reduced to oxidized glutathione
